# Multiplanar strain quantification for assessment of right ventricular dysfunction and non-ischemic fibrosis among patients with ischemic mitral regurgitation

**DOI:** 10.1371/journal.pone.0185657

**Published:** 2017-09-29

**Authors:** Antonino Di Franco, Jiwon Kim, Sara Rodriguez-Diego, Omar Khalique, Jonathan Y. Siden, Samantha R. Goldburg, Neil K. Mehta, Aparna Srinivasan, Mark B. Ratcliffe, Robert A. Levine, Filippo Crea, Richard B. Devereux, Jonathan W. Weinsaft

**Affiliations:** 1 Department of Medicine, Weill Cornell Medical College, New York City, New York, United States of America; 2 Department of Medicine, Columbia University, New York, New York, United States of America; 3 Department of Surgery, University of California San Francisco, San Francisco, California, United States of America; 4 Department of Cardiology, Massachusetts General Hospital, Boston, Massachusetts, United States of America; 5 Department of Cardiology, Università Cattolica del Sacro Cuore, Fondazione Policlinico Universitario A. Gemelli, Rome, Italy; Scuola Superiore Sant'Anna, ITALY

## Abstract

**Background:**

Ischemic mitral regurgitation (iMR) predisposes to right ventricular (RV) pressure and volume overload, providing a nidus for RV dysfunction (RV_DYS_) and non-ischemic fibrosis (NIF). Echocardiography (echo) is widely used to assess iMR, but performance of different indices as markers of RV_DYS_ and NIF is unknown.

**Methods:**

iMR patients prospectively underwent echo and cardiac magnetic resonance (CMR) within 72 hours. Echo quantified iMR, assessed conventional RV indices (TAPSE, RV-S’, fractional area change [FAC]), and strain via speckle tracking in apical 4-chamber (global longitudinal strain [RV-GLS]) and parasternal long axis orientation (transverse strain). CMR volumetrically quantified RVEF, and assessed ischemic pattern myocardial infarction (MI) and septal NIF.

**Results:**

73 iMR patients were studied; 36% had RV_DYS_ (EF<50%) on CMR among whom LVEF was lower, PA systolic pressure higher, and MI size larger (all p<0.05). CMR RVEF was paralleled by echo results; correlations were highest for RV-GLS (r = 0.73) and lowest for RV-S’ (r = 0.43; all p<0.001). RV_DYS_ patients more often had CMR-evidenced NIF (54% vs. 7%; p<0.001). Whereas all RV indices were lower among NIF-affected patients (all p≤0.006), percent change was largest for transverse strain (48.3%). CMR RVEF was independently associated with RV-GLS (partial r = 0.57, p<0.001) and transverse strain (r = 0.38, p = 0.002) (R = 0.78, p<0.001). Overall diagnostic performance of RV-GLS and transverse strain were similar (AUC = 0.93[0.87–0.99]|0.91[0.84–0.99], both p<0.001), and yielded near equivalent sensitivity and specificity (85%|83% and 80%|79% respectively).

**Conclusion:**

Compared to conventional echo indices, RV strain parameters yield stronger correlation with CMR-defined RVEF and potentially constitute better markers of CMR-evidenced NIF in iMR.

## Introduction

Ischemic mitral regurgitation (iMR) predisposes to right ventricular (RV) pressure and volume overload, providing a stimulus for RV dysfunction (RV_DYS_). Echocardiography (echo) is widely used to assess iMR, but performance of different indices as markers of RV_DYS_ and tissue remodeling has not been fully elucidated. Given that RV_DYS_ impacts morbidity and mortality [1,2], validation of established and emerging echo approaches for RV assessment is of substantial importance.

Cardiac magnetic resonance (CMR) enables RV function to be volumetrically quantified, an approach that is highly reproducible and entails no geometric assumptions [3]. CMR allows assessment of myocardial tissue properties [4,5], including non-ischemic fibrosis (NIF)–a marker of response to increased RV afterload that has itself been associated with adverse prognosis [6,7]. Conventional echo RV indices have been compared to CMR in mixed cohorts, for which results have shown limited agreement with volumetric quantification via CMR [8–10].

One potential reason for discordance between CMR and echo may stem from approaches used for RV assessment. Conventional echo methods assess the RV in a single 2D orientation, which can provide limited insight into global RV performance. Recent data by our group and others have shown multiplanar echo quantification–including linear fractional shortening in apical and parasternal long axis (PLAX) views–to yield improved echo assessment of CMR evidenced RV_DYS_ [11–13]. New echo methods enable assessment of myocardial deformation (strain); utility of multiplanar strain imaging for assessment of volumetric RV_DYS_ and NIF-associated RV remodeling by CMR is unknown.

This study examined RV performance among a prospective cohort of patients with iMR undergoing echo and CMR. Goals were to (1) assess prevalence of CMR-evidenced RV_DYS_ (RV ejection fraction [EF<50%]) and septal NIF among patients with iMR; (2) compare the ability of conventional and multiplanar strain RV echo indices to act as markers of CMR-defined RV_DYS_ and NIF.

## Materials and methods

### Study population

Patients were enrolled prospectively from September 2015 to May 2016 as part of an established protocol examining iMR associated remodeling—approximately 20% of patients recruited agreed to enrollment. Eligible patients had documented history of MR (≥mild) and were recruited from those being considered for invasive coronary angiography at Weill Cornell Medical College, in the context of known obstructive coronary artery disease (CAD) or abnormal stress test. All patients had either obstructive CAD based on angiography or prior history of coronary revascularization/myocardial infarction (MI). Patients with primary MR (e.g. prolapse, rheumatic), papillary muscle rupture, prior mitral valve replacement, or contraindications to CMR (NYHA IV, unstable angina, acute MI) or gadolinium (e.g. glomerular filtration rate<30 ml/min/1.73m^2^) were excluded. Clinical indices (including prior MI and coronary revascularization) were attained in a standardized manner using uniform patient questionnaires (administered by research personnel at time of study imaging) and supplemented by review of medical records.

Imaging was performed at Weill Cornell Medical College (New York, NY). The Cornell Institutional Review Board approved this study (Protocol #: 1505016238R002), which was in compliance with the Declaration of Helsinki. Written informed consent was obtained at time of patient enrollment.

### Imaging protocol

Echo and CMR were performed within a 3-day (72-hour) interval using a standardized protocol:

#### Echocardiography

Transthoracic echo was performed using commercial equipment (Philips ie33 [Andover, MA]). Echoes were interpreted by experienced investigators within a high-volume laboratory, for which expertise and reproducibility for quantitative LV and RV indices have been validated and applied in population-based research [12,14,15]. RV systolic function was quantified via TAPSE, RV-S’ and fractional area change (FAC), which were acquired in accordance with consensus guidelines [16]. TAPSE was measured (on M-mode) as the systolic excursion of the lateral tricuspid annulus along its longitudinal plane. RV-S’ was measured (on tissue Doppler) as the peak tricuspid annular longitudinal velocity of excursion. FAC was measured via planimetry of end-diastolic and end-systolic contours in apical 4-chamber orientation. Established cutoffs (TAPSE < 1.6 cm, S’<10 mm/s, FAC< 35%) were used to detect RV_DYS_ by each parameter [16].

**Fig 1:**
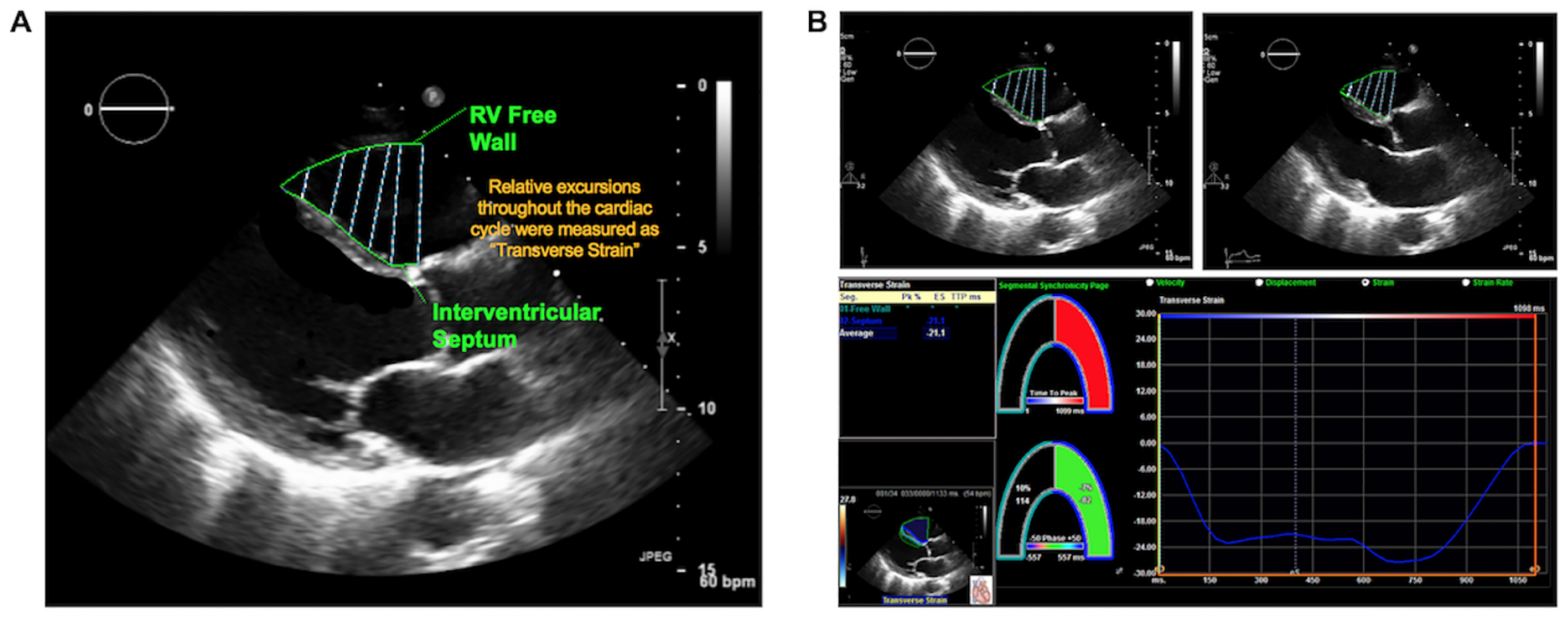
Illustration of analytic method for quantification of transverse strain. **1A**. PLAX images were analyzed via placement of contour lines in the RV free wall and anteroseptum; transverse strain was calculated as relative excursions of the RV free wall in relation to the septum. **1B**. Data output generated from PLAX transverse strain segmentation method. Primary images (end-diastole [left], end-systole [right]) with superimposed strain contours shown on top; resultant strain output and curve shown on bottom.

Strain based indices were also quantified to further assess RV function. To test the utility of multiplanar imaging, strain was measured in two distinct orientations:

- RV longitudinal strain: Global and regional longitudinal strain were measured in 2D apical 4 chamber datasets, for which images were acquired at frame rates of 60–90 Hz. Endocardial tracking points (from tricuspid annulus through RV apex) were placed at end-systole; automated tracking was used to propagate seed points throughout the cardiac cycle and, when required, were manually adjusted by an experienced reader (ADF) to ensure optimal border tracking and deformation curves. Global longitudinal strain (GLS) was calculated as mean of all RV seed points; regional strain was assessed in the inferoseptum and inferior RV free wall (each of which were analyzed as discrete segments).
- RV transverse strain was measured in 2D PLAX. Seed points were placed throughout the superior RV free wall and anteroseptum, encompassing the RV chamber as visualized in PLAX (Fig 1A): Systolic excursions of the RV free wall (in relation to the septum) was measured as “transverse strain” (Fig 1B), which was calculated as a singular discrete variable reflecting the mean of all seed point excursions in the RV superior free wall and anteroseptum.

RV-GLS and transverse strain were assessed in relation to one another as singular indices, each of which reflected global RV excursions in respective orientations (apical 4 chamber, PLAX).

Intra- and inter-observer reproducibility assessments of strain indices (RV-GLS, transverse strain) were determined via blinded repeat analyses of 20 patients. Strain analyses were performed using commercial software (TomTEC [Munich, Germany]) and are reported as absolute values.

Additional analyses were performed to assess ancillary echo indices relevant to RV remodeling. MR severity was measured quantitatively in all patients using regurgitant fraction and/or vena contracta. To account for differences in individual indices, MR severity was also graded in accordance with consensus guidelines [17] using a 5-point (0–4+) scale based on aggregate data yielded by vena contracta, volumetric indices, jet depth as well as mitral and pulmonary vein flow pattern [18,19]. LV systolic function, geometry, and mass were quantified based on linear dimensions in parasternal long axis, consistent with quantitative methods previously validated in necropsy-comparison and population-based outcomes studies [20–23].

#### Cardiac magnetic resonance

CMR was performed using 3.0 Tesla scanners (General Electric, Waukesha, WI). Exams consisted of two components: (1) cine-CMR for geometry/function and (2) delayed enhancement (DE-) CMR for tissue characterization. Cine-CMR was performed using a steady-state free precession sequence. DE-CMR was performed 10–30 minutes after administration of gadolinium (0.2 mmol/kg) using a segmented inversion recovery sequence, with inversion time tailored to null viable myocardium. Cine- and DE-CMR were obtained in matching LV short and long-axis planes. LV infarct size was measured on DE-CMR, for which transmural extent and regionality was scored using a 17-segment model: Infarct size was graded based on transmural extent of hyperenhancement; global infarct size (% LV myocardium) was calculated by summing all segmental scores (weighted by the midpoint of hyperenhancement range) and dividing by total number of regions [24,25].

**Fig 2:**
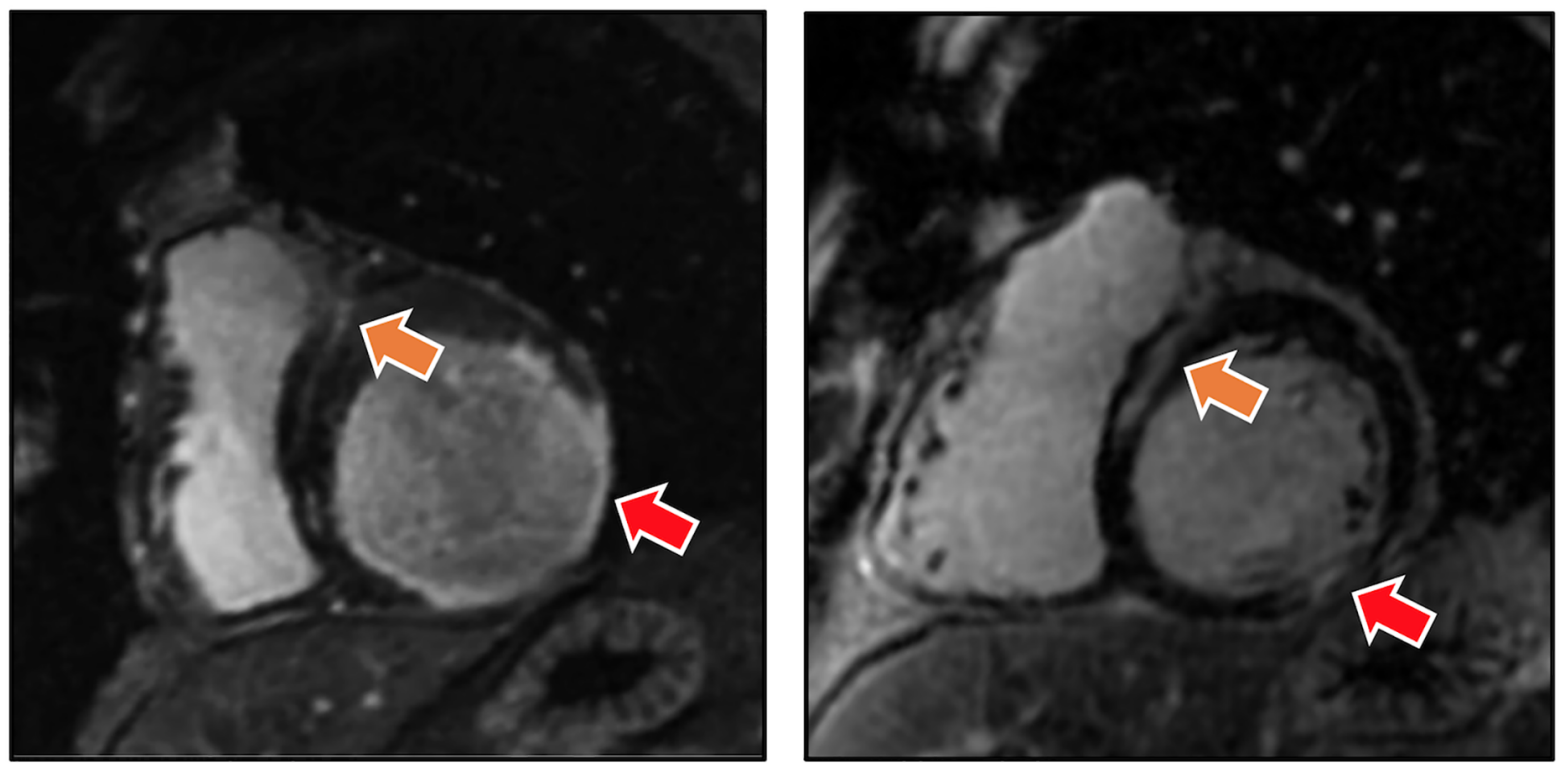
Representative examples of ischemic (red arrows) and non-ischemic (orange arrows) patterns of late gadolinium enhancement demonstrated by DE-CMR among patients with iMR and RV_DYS_. Note concomitant NIF (localized to the mid-myocardial aspect of the interventricular septum) and CAD pattern transmural MI.

DE-CMR was also used to identify NIF, which was defined as localized hyperenhancement in the mid myocardial or epicardial aspect of the basal to mid inter-ventricular septum (Fig 2), in accordance with prior research by our group and others [26–28]. Cine-CMR was used to assess RV and LV geometry/function: End-diastolic and end-systolic chamber volumes were measured in contiguous short axis images, with results used to calculate EF. Cine-CMR quantified RVEF was employed as the reference standard for RV_DYS_, which was defined using an established binary cutoff (RVEF< 50%) [11,29–31]. CMR analyses were performed by an experienced reader (JWW), for whom high reproducibility for both LV and RV indices has been documented [12,32,33].

### Statistical analysis

Continuous variables (expressed as mean±standard deviation) were compared using Student’s t-tests. Categorical variables were compared using Chi-square or, when fewer than 5 expected outcomes per cell, Fisher’s exact test. Ordinal comparisons (i.e. MR grade) were performed using the Mann-Whitney U test. Correlation coefficients, as well as univariable and multivariable regression analyses were used to evaluate associations between continuous variables. Inter-observer and intra-observer agreement between methods was assessed using Bland and Altman analysis, including mean difference and limits of agreement between measurements (mean±1.96 SD). Percent changes were calculated by means of the following formula (with the larger mean always used in the denominator):

$$\frac{\left(\text{mean}\;\text{value}\;\text{Group}\;1\right)-\left(\text{mean}\;\text{value}\;\text{Group}\;2\right)}{\left(\text{mean}\;\text{value}\;\text{Group}\;1\right)}*100$$

Cohen’s D test was used to measure “effect size”. Two- sided p<0.05 was considered indicative of statistical significance. Statistical calculations were performed using SPSS 20.0 (SPSS Inc, Chicago, IL).

## Results

### Population characteristics

The population comprised 73 patients with iMR who underwent CMR and echo within mean interval of 0.2±0.6 days (96% same day). Over one third (36%) of patients had RV_DYS_ (EF<50%) as defined by the reference standard of CMR: Among affected patients, RV_DYS_ magnitude varied (RVEF<30%: 19% [n = 5] | 30–40%: 23% [n = 6] | 41–49%: 58% [n = 15]). Obstructive CAD was confirmed via invasive angiography in 95% (69/73) of patients (84% multivessel CAD)–all remaining patients had a history of prior PCI and/or ECG/imaging localized inferolateral MI. Clinically reported MI was present in nearly 2/3 (64%) of patients (mean interval 2.6±4.2 years prior to CMR). MI incidence as identified by DE-CMR (i.e. CAD pattern infarction) was slightly higher (83%) than that of clinical MI: Nearly a third of patients in the overall cohort (29%) had multiple DE-CMR evidenced infarcts in distinct coronary arterial territories.

Table 1 details clinical and imaging characteristics of the population, as well as comparisons between patients with and without RV_DYS_. As shown, patients with RV_DYS_ were similar with respect to CAD risk factors, but were more likely to require heart failure medications such as ACE inhibitors or loop diuretics, consistent with larger MI size and higher pulmonary artery (PA) pressure (all p<0.05). Both CMR and echo demonstrated patients with RV_DYS_ to have more advanced adverse LV remodeling, whether measured by LVEF or LV chamber size (both p<0.05). Consistent with this, MR severity was strongly linked to RV contractile impairment, as evidenced by nearly a 2.5-fold increase in prevalence of advanced (≥moderate) MR among patients with, compared to those without, RV_DYS_ (73% vs. 30%, p = 0.001). In multivariate analysis, RV_DYS_ was independently associated with advanced MR (OR 6.1 [95% CI 1.7–21.4]; p = 0.005) even after controlling for magnitude of CMR-defined LV dysfunction (OR 2.0 per 10-point decrement in LVEF [CI 1.4–2.6] p< 0.001) (model χ^2^ = 32.95, p<0.001) (Table 2). Substitution of echo derived LVEF in the model yielded similar results, again showing RV_DYS_ to be associated with advanced MR (OR 5.5 [95% CI 1.6–19.3]; p = 0.008) independent of global LV dysfunction (OR 2.0 per 10-point decrement in LVEF [95% CI 1.5–2.6]; p< 0.001) (model χ^2^ = 32.98, p< 0.001).

**Table 1:** Clinical and imaging characteristics.

	Overall (n = 73)	RV_DYS_ − (n = 47)	RV_DYS_ + (n = 26)	p
**CLINICAL**				
**Age (years)**	68.3±9.9	68±9	70±11	0.38
**Male gender**	61 (84%)	37 (79%)	24 (92%)	0.19
**Body Surface Area**	1.9±0.2	1.9±0.3	1.9±0.2	0.63
**Coronary Artery Disease Risk Factors**				
Hypertension	58 (80%)	39 (83%)	19 (73%)	0.32
Hypercholesterolemia	55 (75%)	37 (79%)	18 (69%)	0.37
Diabetes Mellitus	39 (53%)	24 (51%)	15 (58%)	0.59
Tobacco Use	43 (59%)	26 (55%)	17 (65%)	0.40
Family History	17 (23%)	11 (23%)	6 (23%)	0.98
**Prior Coronary Revascularization (PCI or CABG)**	58 (80%)	36 (77%)	22 (85%)	0.42
**Prior CABG**	26 (36%)	15 (32%)	11 (42%)	0.38
Prior Myocardial Infarction*	47 (64%)	29 (62%)	18 (69%)	0.61
**Clinical Symptoms**				
Angina	44 (60%)	26 (55%)	18 (69%)	0.25
Dyspnea	58 (80%)	36 (77%)	22 (85%)	0.42
**Cardiovascular Medications**				
Beta-blocker	60 (82%)	38 (81%)	22 (85%)	0.76
ACE-Inhibitor or ARB	42 (58%)	22 (47%)	20 (77%)	**0.013**
Loop diuretic	26 (36%)	11 (23%)	15 (58%)	**0.003**
Statins	59 (81%)	38 (81%)	21 (81%)	0.99
Aspirin	62 (85%)	41 (87%)	21 (81%)	0.51
Thienopyridine	32 (44%)	20 (43%)	12 (46%)	0.81
**CARDIAC MAGNETIC RESONANCE**				
**Left Atrial / Mitral Apparatus Remodeling**				
Left atrial area (cm^2^)	27.7±7.0	26.3±7.0	30.4±6.2	**0.014**
Left atrial diameter (cm)	4.3±0.7	4.1±0.5	4.6±0.7	**0.001**
Mitral valve tenting area– 4 chamber (cm^2^)	1.8±0.9	1.6±0.9	2.0±0.8	**0.037**
**Left Ventricle**				
Ejection fraction (%)	42.4±16.0	48.7±14.4	31.1±12.2	**<0.001**
Stroke volume (ml)	79.4±24.7	85.0±24.5	69.2±22.2	**0.008**
End-diastolic volume (ml)	202.7±61.9	186.3±60.1	232.4±54.6	**0.002**
End-systolic volume (ml)	123.4±63.4	101.3±56.7	163.3±55.5	**<0.001**
Myocardial mass (g)	160.0±44.3	153.1±48.4	172.7±32.7	0.07
Sphericity index	0.5±0.1	0.45±0.09	0.54±0.09	**<0.001**
**Myocardial Infarction**°	60 (83%)	36 (78%)	24(92%)	0.19
Global MI Size (% LV myocardium)	9.8±9.2	8.0±8.9	13.1±9.0	**0.02**
Anterior MI (% myocardium)	1.7±2.9	1.6±3.1	1.8±2.6	0.74
Lateral MI (% myocardium)	3.5±5.4	3.0±5.3	4.6±5.7	0.22
Inferior MI (% myocardium)	3.1±4.6	2.4±4.1	4.4±5.1	0.08
Multiple Myocardial Infarctions	21 (29%)	11 (24%)	10 (39%)	0.19
**Right Ventricle**				
Ejection fraction (%)	51.7±11.9	58.8±6.6	38.9±7.9	**<0.001**
Stroke volume (ml)	74.8±20.8	79.9±21.9	65.6±14.9	**0.002**
End-diastolic volume (ml)	151.5±51.1	138.7±43.0	174.7±56.9	**0.003**
End-systolic volume (ml)	76.5±42.6	58.4±23.7	109.1±49.7	**<0.001**
**ECHOCARDIODIOGRAPHY**				
**Mitral Regurgitation**				
Regurgitant Fraction (%)	39.2±14.6	35.9±14.8	43.9±13.3	**0.03**
Vena Contracta	0.3±0.1	0.3±0.1	0.4±0.1	**0.02**
Mitral regurgitation severity (grade 1–4)	40(55%) | 22 (30%) 7(10%) | 4(6%)	33 (70%) | 10(21%) 3(6%) | 1(2%)	7(27%) | 12(46%) 4(15%) | 3(12%)	**<0.001**
Advanced mitral regurgitation (≥moderate)	33 (45%)	14 (30%)	19 (73%)	**0.001**
**Left Ventricle**				
Ejection fraction (%)	41.9±15.9	48.1±14.8	30.6±10.8	**<0.001**
End-diastolic diameter (cm)	5.9±0.6	5.7±0.6	6.2±0.6	**0.004**
End-systolic diameter (cm)	4.7±0.9	4.4±0.8	5.3±0.7	**<0.001**
Myocardial mass (g)	212.9±69.2	207.8±74.6	222.2±58.5	0.40
**Pulmonary Arterial Pressure**^§^ (mmHg)	38.0±16.1	34.3±14.5	44.4±16.9	**0.02**
**Pulmonary Hypertension**^§^	30 (41%)	15 (32%)	15 (58%)	**0.032**

Data (continuous indices) presented as mean ±standard deviation.

*Myocardial infarction classified based on clinical history/prior medical records

°Assessed in 99% of population (n = 1; gadolinium not administered due to IV malfunction)

^§^Available in 78% of population (pulmonary hypertension defined as PA systolic pressure > 35mmHg)

**Table 2:** RV_DYS_ in relation to MR and LV_DYS_.

	Univariate Regression		Multivariate Logistic Regression *Model χ*^*2*^ = *32*.*95*, *p<0*.*001*	
	Odds Ratio (95% Confidence Interval)	P	Odds Ratio (95% Confidence Interval)	P
**Advanced MR** (≥moderate)	6.4 (2.2–18.6)	**0.001**	6.1 (CI 1.7–21.4)	**0.005**
**LVEF** (per 10% decrement)	2.0 (1.5–2.6)	**<0.001**	2.0 (CI 1.4–2.6)	**<0.001**

### Apical echocardiographic indices of RV function

RV volumetric measurements were compared to echo strain and conventional indices of RV performance as measured in apical 4-chamber orientation: Nearly all exams (93%) yielded full datasets inclusive of TAPSE, RV-S’, FAC, and RV-GLS. RV-GLS, in particular, was obtained in 97% (71/73) of exams.

**Fig 3:**
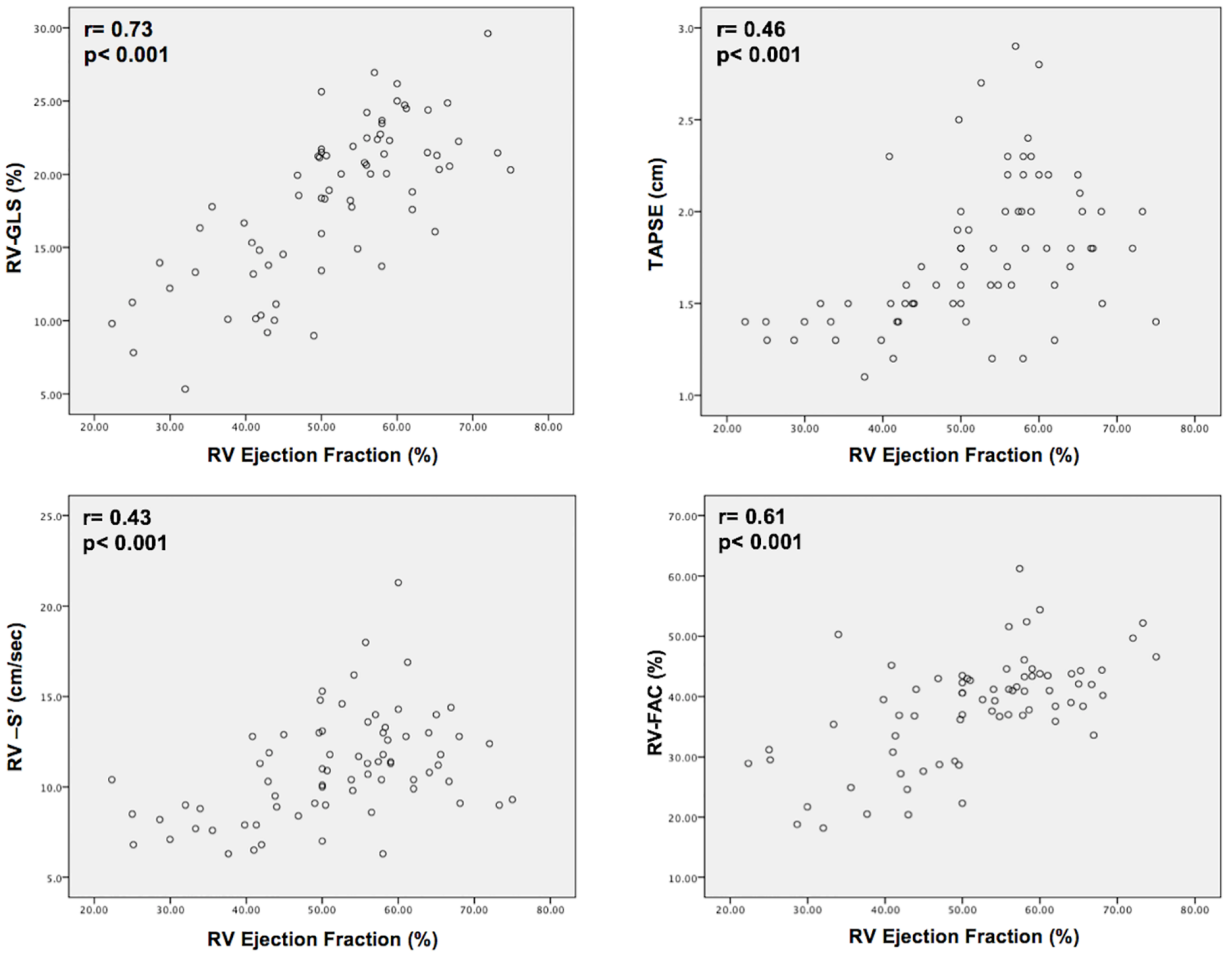
Scatter plots comparing conventional echo RV parameters in relation to CMR RVEF. Whereas all echo indices correlated with volumetric RVEF (p<0.001), RV-GLS (upper left) yielded higher correlations than did TAPSE, RV-S’ or FAC.

Table 3 reports echo-derived variables, including comparisons between patients with and without CMR defined RV_DYS_. As shown, whereas all echo variables differed significantly between groups (all p≤0.001), percent changes were larger for longitudinal strain compared to conventional indices. For example, RV-GLS was 1.6-fold lower among patients with RV_DYS_, whereas TAPSE, RV-S’ and FAC yielded differences of 1.3–1.4 fold. Consistent with this, data shown in Fig 3 demonstrate that correlations between CMR RVEF and echo RV-GLS (r = 0.73) were higher than those yielded by TAPSE (r = 0.46), RV-S’ (r = 0.43) or FAC (r = 0.61) (all p<0.001).

**Table 3:** Conventional echo RV functional indices.

	Overall (n = 73)	RV_DYS_ − (n = 47)	RV_DYS_ + (n = 26)	P	%Δ	Effect Size
TAPSE (cm)	1.8±0.4	1.9±0.4	1.5±0.3	**<0.001**	**21.1**	**1.1**
RV-S’ (cm/sec)	11.0±2.9	12.0±2.8	9.3±2.3	**<0.001**	**22.5**	**1.1**
FAC (%)	38.2±8.8	42.2±6.1	31.1±8.4	**<0.001**	**26.3**	**1.5**
RV global longitudinal strain (%)	18.3±5.3	21.2±3.4	13.3±4.2	**<0.001**	**37.3**	**2.1**
RV free wall longitudinal strain (%)	18.1±6.6	21.5±4.3	12.3±5.8	**<0.001**	**42.8**	**1.8**
RV septal longitudinal strain (%)	11.1±6.8	13.2±6.5	7.6±5.9	**0.001**	**42.4**	**0.9**

### CMR tissue characterization in relation to echo-based RV assessment

Despite angiographic-evidenced CAD, tissue characterization via CMR demonstrated septal NIF to be common in patients with iMR: NIF was present in 24% of the population, and was 8-fold more common in patients with RV_DYS_ (54% vs. 7%; p<0.001). Patients with NIF had more advanced RV_DYS_ and adverse remodeling on cine-CMR, as evidenced by lower RVEF (41.4±9.6 vs. 54.6±10.7%, p< 0.001), larger chamber size (RV end diastolic volume: 177.2±61.8 vs. 144.4±45.2 ml, p = 0.02), and higher PA pressure (51.7±21.9 vs. 33.6±10.7 mmHg, p<0.001). Regarding distribution, NIF most commonly localized to the anteroseptum (anteroseptum only: 59%|inferoseptum only: 12%|anterior and inferoseptum: 29%).

Given that NIF commonly localized to the anteroseptum (not encompassed via 4 chamber orientation), strain analysis was also performed in PLAX (an orientation that enables assessment of anteroseptal transverse displacement). PLAX derived RV transverse strain was obtainable in 92% (67/73) of exams.

**Fig 4:**
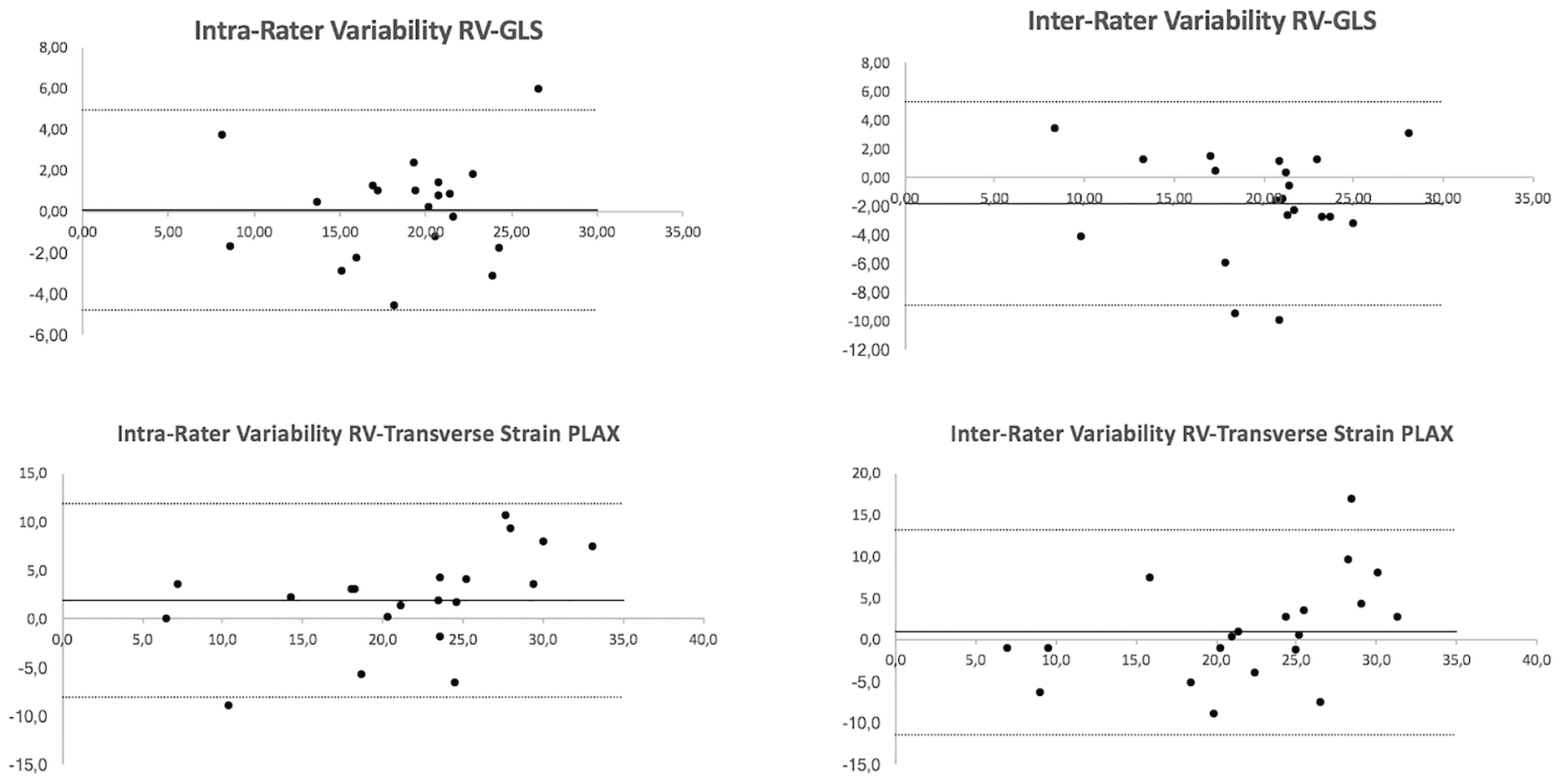
Bland-Altman plots demonstrating magnitude of intra- and inter-observer agreement for RV-GLS (top) and transverse strain (bottom).

Reproducibility was good for both RV-GLS and transverse strain, with small mean differences (intra-observer: 0.08±2.49 vs. 1.9±5.08, respectively; inter-observer: -1.81±3.64 vs. 0.9±6.25, respectively) although limits of agreement were wider for transverse strain than for RV-GLS (intra-observer: -8.0 to 11.9 vs. -4.8 to 4.9, respectively; inter-observer: -11.4 to 13.1 vs. -8.9 to 5.3, respectively) (Fig 4).

**Fig 5:**
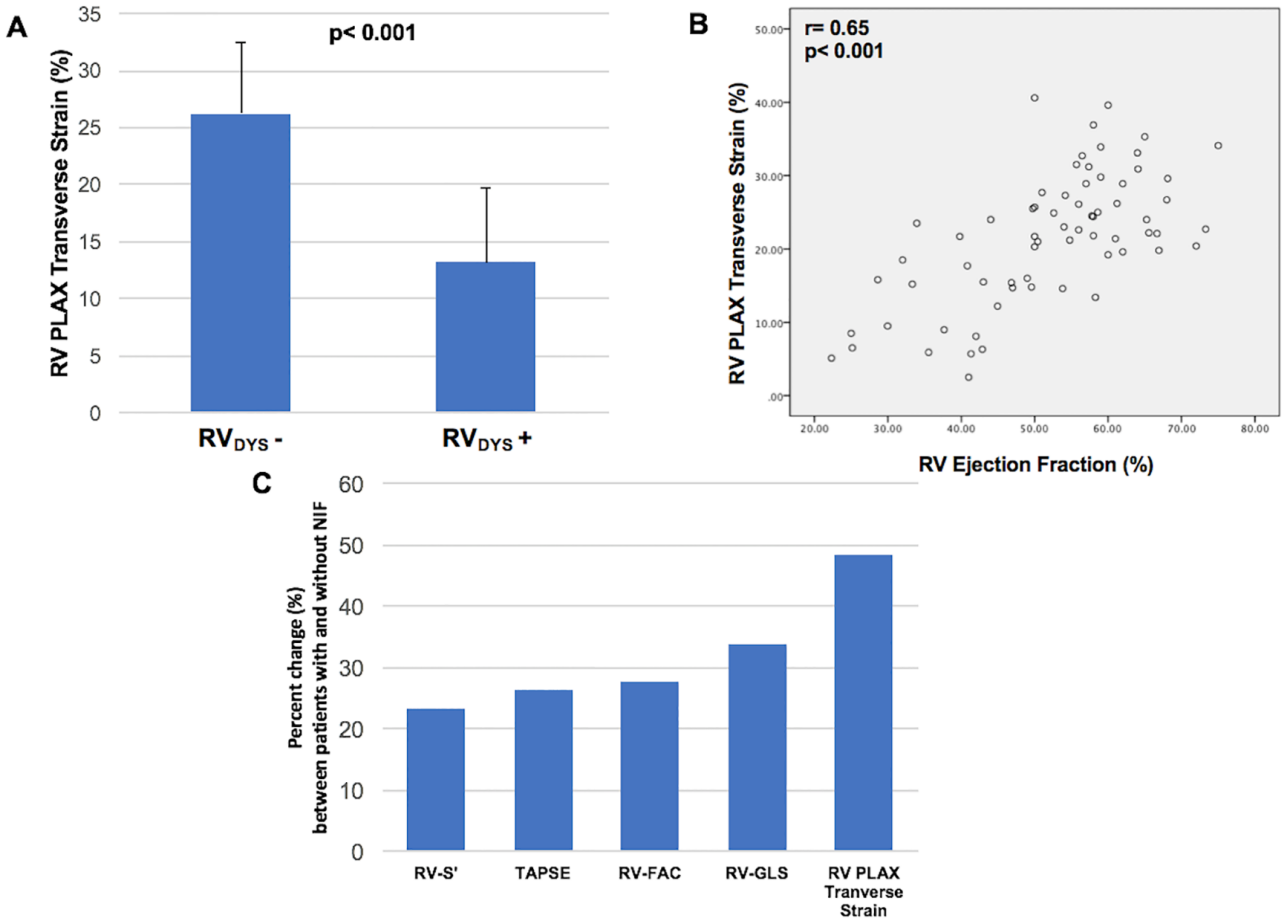
**5A**. PLAX transverse strain values among patients with and without CMR-defined RV_DYS_ (EF<50%). Data reported as mean ± standard deviation. **5B**. Correlation between transverse strain and CMR-RVEF. **5C**. Percent change (%) between patients with and without NIF as yielded by respective echo RV parameters. Note larger percent change for transverse strain compared to other echo indices.

As shown in Fig 5A, PLAX transverse strain was lower among iMR patients with, vs. those without, CMR-defined RV_DYS_ (13.2±6.6 vs. 26.2±6.2%, p< 0.001)–magnitude of difference in terms of “effect size” was equivalent to that yielded by RV-GLS (2.0 vs 2.1, respectively). Consistent with this, data shown in Fig 5B demonstrate that transverse strain correlated with CMR RVEF (r = 0.65; p< 0.001). RV-GLS and PLAX transverse strain yielded similar overall performance for diagnostic assessment of NIF as identified by DE-CMR (AUC: 0.87 [GLS], 0.88 [transverse]; both p<0.001). Of note, whereas all echo RV indices differed significantly between patients with and without NIF (all p<0.01), percent change was larger for transverse strain as compared to RV-GLS or conventional indices (Fig 5C) (Table 4). In multivariate analysis, RVEF on CMR was independently associated with both echo-quantified RV-GLS (partial correlation coefficient [r] = 0.57, p<0.001) as well as transverse strain (r = 0.38, p = 0.002) (model R = 0.78, p<0.001) (Table 5).

**Table 4:** Echo strain indices stratified between patients with and without CMR-evidenced NIF*.

	NIF − (n = 55)	NIF + (n = 17)	P	%Δ	Effect Size
TAPSE (cm)	1.9±0.4	1.4±0.1	**<0.001**	26.3	1.7
RV-S’ (cm/sec)	11.6±2.9	8.9±1.8	**0.001**	23.3	1.1
FAC (%)	40.9±7.5	29.5±7.2	**<0.001**	27.9	1.6
RV global longitudinal strain (%)	20.0±4.6	13.2±3.9	**<0.001**	34.0	1.6
RV free wall longitudinal strain (%)	20.2±5.8	11.6±5.1	**<0.001**	42.6	1.6
RV septal longitudinal strain (%)	12.3±6.7	7.3±6.0	**0.006**	40.7	0.8
RV transverse strain (%)	24.0±7.6	12.4±6.4	**<0.001**	48.3	1.7

* Assessed in 99% of population (n = 1; gadolinium not administered due to IV malfunction)

**Table 5:** CMR RVEF in relation to echo global longitudinal and transverse strain.

	Univariate Correlations		Multivariate Linear Regression *Model R = 0*.*78*, *p<0*.*001*	
	Correlation Coefficients	P	Partial Correlation	P
**Global Longitudinal Strain**	0.73	**<0.001**	0.57	**<0.001**
**Transverse Strain**	0.65	**<0.001**	0.38	**0.002**

### Diagnostic performance of echo indices for CMR defined RV dysfunction

**Fig 6:**
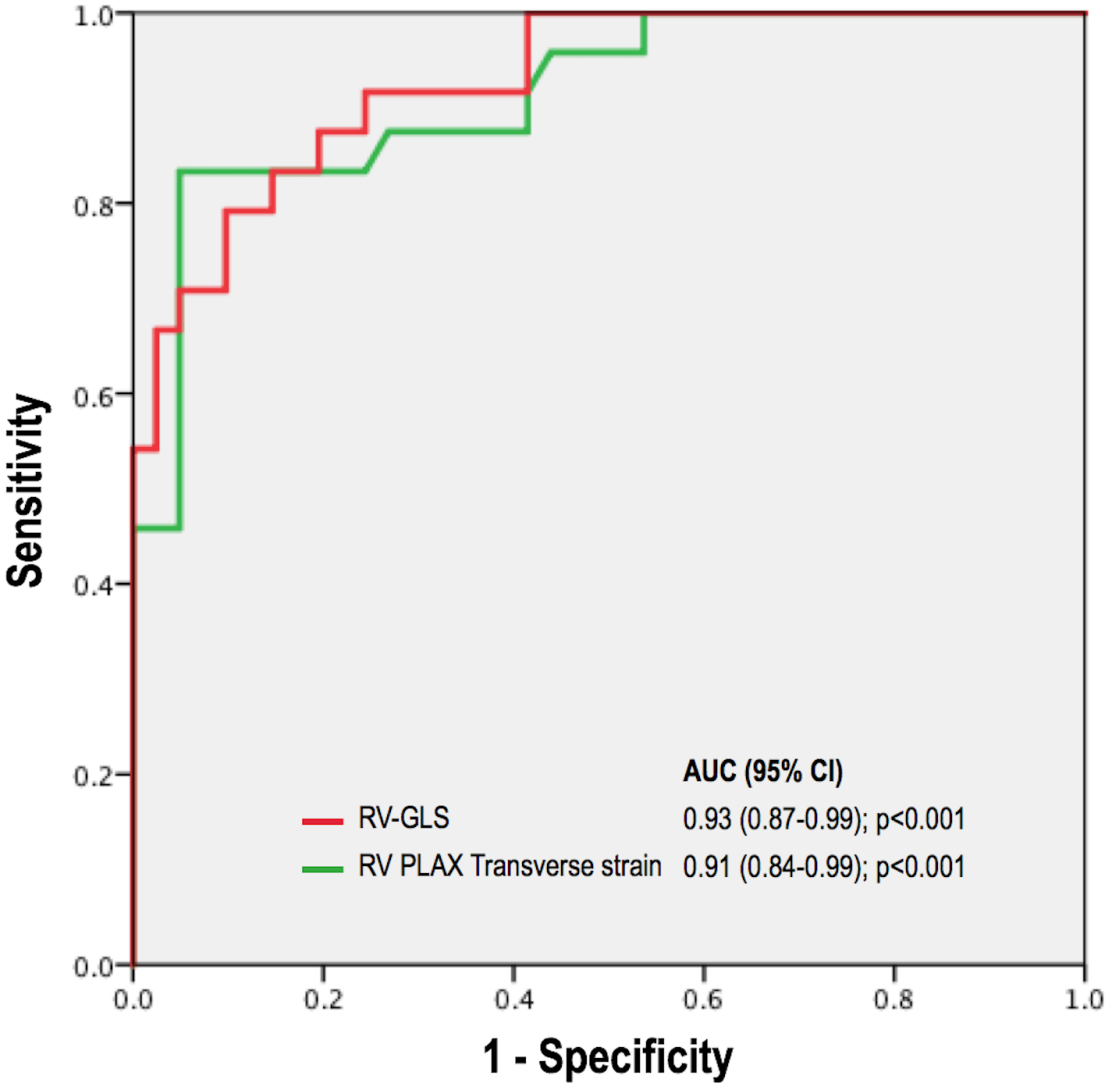
Superimposed receiver operating curve (ROC) analyses for RV-GLS and transverse strain, demonstrating high overall diagnostic performance (AUC > 0.90) for both strain indices.

Fig 6 provides superimposed ROC analyses for RV-GLS and transverse strain. As shown, overall diagnostic performance for each parameter was of similarly high magnitude (AUC 0.93 [0.87–0.99], p<0.001 and 0.91 [0.84–0.99], p<0.001, respectively). Using a matched specificity cutoff of 80%, RV-GLS yielded a sensitivity of 85% (cut-off value for RV-GLS: 18%). Similarly, transverse strain yielded a sensitivity of 83% (cut-off value for transverse strain: 21%).

Diagnostic performance parameters of strain and conventional indices are reported in Table 6. As shown, transverse strain yielded good overall accuracy (81%), which was similar to RV-GLS and conventional echo parameters as acquired in apical 4-chamber orientation (76–85%).

**Table 6:** Diagnostic test performance of both apical and PLAX echo indices of RV function.

	Sensitivity	Specificity	PPV	NPV	Accuracy
TAPSE*	76%	85%	73%	87%	82%
RV-S’*	71%	81%	65%	83%	76%
FAC*	65%	96%	89%	83%	85%
RV global longitudinal strain**	85%	80%	71%	90%	82%
RV PLAX transverse strain**	83%	79%	69%	89%	81%

* Tested using cutoffs included in consensus guidelines (TAPSE <1.6 cm; RV-S’<10 cm/sec; FAC<35%) [16]

**Tested using cutoffs (RV-GLS 18%, transverse strain 21%) derived from ROC analysis (Fig 6).

## Discussion

This study yields new insights regarding RV pathophysiology in patients with iMR, as well as novel echo methods for assessment of RV_DYS_. Major findings are as follows: (1) Among a cohort of patients with iMR, RV_DYS_ was common and strongly associated with LV dilation and contractile dysfunction (all p<0.05) measured by both CMR and echo. (2) Conventional and longitudinal strain indices differed between patients with and without CMR-evidenced RV_DYS_, but RV-GLS yielded higher correlations with RVEF (r = 0.73) than did FAC, TAPSE, and RV-S’ (r = 0.43–0.61; all p<0.001). Transverse strain yielded similar correlation with CMR RVEF (r = 0.65; p<0.001) as did RV-GLS, as well as similar overall diagnostic performance for RV_DYS_ (EF<50%) (AUC 0.91 [0.84–0.99], 0.93 [0.87–0.99] respectively [both p<0.001]). (3) Despite epicardial CAD, patients with iMR commonly had CMR-evidenced NIF (24%), which was 8-fold more prevalent among those with RV_DYS_ (54% vs. 7%; p<0.001). NIF was associated with lower RV-GLS (measured in 4-chamber orientation) and transverse strain (measured in PLAX) (both p<0.001). Percent change between patients with and without NIF was highest for RV transverse strain (48.3%) compared to RV-GLS (34.0%), TAPSE (26.3%), RV-S’ (23.3%), and FAC (27.9%).

Our finding of an association between volumetric RVEF and transverse strain–a measure of RV free wall contractility–builds upon a growing body of literature concerning utility of multiplanar echo for RV assessment. 3D echo has been shown to improve RV quantification compared to conventional echo approaches derived from data acquired in a single orientation, and yield improved agreement with CMR [34–36]. Utility of quantitative RV assessment in multiple orientations has also been shown using 2D echo. Among 272 CAD patients undergoing CMR and echo, our group showed echo linear RV dimensions in multiple orientations to increase in proportion to CMR-evidenced RV chamber volumes [12]. This concept was subsequently tested among patients with and without biventricular heart failure, in whom CMR-quantified RVEF was independently associated with linear fractional shortening in PLAX (partial correlation r = 0.50, p<0.001) and apical 4 chamber orientation (r = 0.40, p<0.001) [11]. Our current study extends on this using strain–a newly available quantitative method that assesses aggregate contractility in a given plane rather than in an isolated linear dimension. Paralleling findings of our prior studies, RVEF was independently associated with both transverse (partial correlation r = 0.38, p = 0.002) and global strain (r = 0.57, p<0.001; model R = 0.78, p<0.001). These data support the concept that RV_DYS_ reflects a global process in patients with iMR (due to regurgitation-associated increments in RV afterload) that can be well assessed via multiplanar imaging.

Magnitude of correlation between strain indices and RVEF in our cohort is consistent with that reported in prior studies. However, prior studies have varied with respect to interval between testing, and no prior study has focused on RV strain in the context of MR. Among 135 post-MI patients, Lemarie et al. reported that RV-GLS yielded a higher correlation with CMR RVEF (r = 0.46) than did TAPSE (r = 0.26), RV-S’ (r = 0.18), or FAC (r = 0.37). [37] Prior reports on patients with advanced LV_DYS_ have demonstrated stronger correlations between echo strain and CMR. For example, among 57 patients with ischemic cardiomyopathy (LVEF<40%), Park et al. reported that RV-GLS correlated well with CMR RVEF (r = 0.80) [38], consistent with our findings (r = 0.73). In our study, improved correlations with CMR RVEF yielded by strain were not accompanied by marked increments in diagnostic performance for RV_DYS_ using a binary threshold of RVEF <50%. For example, RV-GLS yielded sensitivity and specificity of 85% and 80% respectively, whereas respective values for TAPSE were 76% and 85%. We speculate that whereas strain provides incremental utility as a continuous index of RVEF (accounting for improved correlation coefficients), conventional indices provide a reasonable binary means of discriminating between patients with and without RV_DYS_. It is also important to note that all echoes in our study were specifically performed for research purposes, and were thus of higher quality than might be anticipated in general clinical practice.

Beyond RV function, it is important to note that whereas our cohort consisted of iMR patients with epicardial CAD, 24% of all patients (including 54% of those with RV_DYS_) had NIF identified by CMR tissue characterization. To the best of our knowledge, this is the first study to assess the impact of NIF on RV_DYS_ in the setting of patients with iMR as well as its relationship with RV strain. Our finding of an association between NIF and iMR is consistent with prior basic science studies showing MR to induce up-regulation of pro-inflammatory cell-signaling pathways promoting adverse remodeling [39,40]. Up-regulation of pro-fibrotic signaling pathways has also been suggested as a potential contributing factor in the pathophysiology of NIF. In a rat model of hypoxia-induced pulmonary hypertension, McKenzie et al. reported that expression of atrial natriuretic peptide—a vasodilatory peptide secreted in pathologic conditions of increased myocardial load—was most prominent in the RV insertion points and the interventricular septum (corresponding to NIF location on CMR) [41]. Evidence that NIF might contribute to ventricular failure (rather than being a sequela) has recently been suggested in patients with LV_DYS_. Among patients with non-ischemic cardiomyopathy, Taylor et al. demonstrated NIF to be associated with decreased global LV circumferential strain (p = 0.004) as measured via feature tracking CMR [42]. Our results indicate that NIF is similarly paralleled by RV strain impairments as measured via echo speckle tracking.

Regarding mechanistic links between NIF, RV_DYS_ and iMR, it should be noted that prior studies have shown NIF to be associated with RV_DYS_ and adverse RV remodeling–including work by our group which has linked NIF to increased RV wall stress [26]. These data suggest a mechanism whereby iMR results in increased PA pressure, which produces afterload-associated decrements in RV contractile function as well as NIF. It is also known that iMR can result from (and contribute to) LV chamber dilation–a known cause of increased LV wall stress that has itself been associated with NIF [28]. Increased septal stiffness as can result from NIF would be expected to further impede RV mechanics, resulting in further decrements in RV contractility as manifest via both decreased RVEF and impaired strain. Taken together with prior literature, our data suggest that NIF may be both a consequence of and contributor to adverse remodeling irrespective of ventricular chamber involvement.

Several limitations should be noted. First, this study assessed RV physiology in patients with iMR and thus it is uncertain whether results can be extrapolated to other etiologies of MR. On the other hand, a primary sequela of MR (irrespective of etiology) is right-sided pressure and volume overload–known stimuli for RV_DYS_ as well as NIF. Second, transverse strain was measured in PLAX rather than a tailored RV orientation, and regional RV strain in PLAX was not quantified as has been the case for apical 4-chamber derived RV strain. Nevertheless, to the best of our knowledge, this is the first study to assess RV deformation in an orientation different than apical 4-chamber. PLAX, in particular, is a well-established standardized orientation encompassed in nearly all echo exams, and allows for RV evaluation in a plane other than apical 4 chamber (which assesses the inferior RV). Given that apical 4 chamber derived measurements are increasingly being applied for analysis of segmental RV strain [43], future research is needed to test utility of regional strain assessment as quantified in PLAX. Third, it should be noted that our study tested utility of multiplanar imaging using conventional 2D data, rather than 3D echo. Despite this, whereas 3D echo has been shown to yield improved RV assessment [34–36], its widespread use remains limited due to both commercial factors as well as technical challenges, emphasizing the continued importance of 2D echo approaches for both clinical purposes and population-based research. It is also important to note that all echoes in our study were performed for research purposes, and were thus of higher quality than might be anticipated in general clinical practice such that study results reflect a potential “best case scenario” with respect to performance of both transverse and longitudinal strain. It should also be noted that our study included patients who had undergone CABG. Given that prior literature has suggested that cardiac surgery itself can transiently impact RV function [44,45], it is possible that physiologic basis of RV_DYS_ in this subgroup differed from the remainder of our population and that heterogeneity in prior revascularization confounded our results. Moreover, our study population underwent imaging at a single center, and clinical status or prognostic outcomes in this cohort were not tested in relation to either RV function or NIF.

In conclusion, this study demonstrates that RV_DYS_ in patients with iMR is commonly associated with NIF on CMR and provides proof of concept concerning utility of multiplanar strain assessment for evaluation of RV_DYS_ and altered tissue substrate. Further studies are warranted to elucidate novel structural risk factors for iMR itself, whether NIF or strain based indices distinguish between iMR patients with persistent or reversible RV functional impairment, as well as prognostic implications of RV_DYS_ among patients with iMR.
